# Cluster headache associated with a clinically non-functioning pituitary adenoma: a case report

**DOI:** 10.1186/1752-1947-8-451

**Published:** 2014-12-20

**Authors:** Bengt Edvardsson

**Affiliations:** 3 Department of Clinical Sciences, Lund University, Lund, S-221 85 Sweden; 4 Department of Neurology, Skane University Hospital, Lund, S-221 85 Sweden

**Keywords:** Cluster headache, Neuroimaging, Pituitary adenoma, Secondary, Symptomatic

## Abstract

**Introduction:**

Cluster headache belongs to a group of primary headache entities: the trigeminal autonomic cephalalgias. Cluster headache is the most common variant. The headache is usually severe and it is also associated with autonomic symptoms. Secondary causes of cluster headache have been reported, such as intracranial artery aneurysms and tumors. The question of when to carry out neuroimaging in patients with cluster headache is yet unsettled. To the best of the author's knowledge, cluster headache associated with a clinically non-functioning pituitary adenoma (chromophobe adenoma) has not been described. This case report describes the case of a man with cluster headache where the evaluation showed a clinically non-functioning pituitary adenoma.

**Case presentation:**

This case involved a 49-year-old Caucasian man who presented with a one-month history of side-locked attacks of pain located in the right orbit. His symptoms fulfilled the criteria for cluster headache and a diagnosis of cluster headache was made. The patient responded to symptomatic treatment. Enhanced magnetic resonance imaging showed a pituitary adenoma. Further evaluations including hormonal screening revealed a clinically non-functioning pituitary adenoma (chromophobe adenoma). After surgery to remove the tumor, his headache attacks resolved totally.

**Conclusion:**

Tumors have been reported in patients with cluster headache whose clinical attacks are identical to genuine cluster headache. A clinically non-functioning pituitary adenoma can present as cluster headache. This case emphasizes the need of imaging procedures in patients with cluster headache. Contrast-enhanced magnetic resonance imaging including the sella turcica should always be done in patients with cluster headache.

## Introduction

Cluster headache (CH) belongs to a group of primary headache entities, the trigeminal autonomic cephalalgias. CH is the most common variant. Studies have demonstrated that CH has a lifetime prevalence of 0.12%. The headache is usually severe and is associated with autonomic symptoms. CH is more common in men and typically manifests itself between the third and fifth decades of life. The question of when to carry out neuroimaging in patients with CH is yet unsettled. Secondary causes of CH have been reported in many cases, such as intracranial artery aneurysms and tumors (symptomatic CH) [1]. The prevalence of symptomatic CHs is not identified because of a lack of prospective population-based studies. CH associated with pituitary adenoma has been described [2]. To the best of the author's knowledge. CH associated with a clinically non-functioning pituitary adenoma (chromophobe adenoma) has not been described. This case report describes a man with typical CH in the setting of a clinically non-functioning pituitary adenoma.

## Case presentation

**Figure 1:**
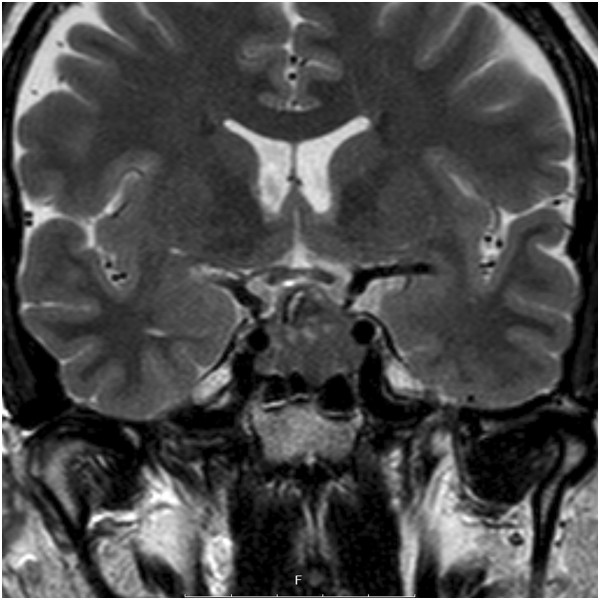
**Coronal T2-weighted magnetic resonance imaging of the sella, showing a 20** **×21** **×17mm pituitary adenoma.** No clear cavernous sinus invasion can be demonstrated.

A 49-year-old Caucasian man presented with a one-month history of side-locked attacks of excruciatingly severe stabbing and boring right-sided pain located in his orbit. The attacks were associated with nasal obstruction, conjunctival injection, restlessness and migrainous features such as nausea, photophobia and phonophobia. No continuous background pain was identified. The duration of the attacks was about 20 to 30 minutes with a frequency of two to three per 24 hours. The attacks were stereotypical, being exclusively right-sided with severe agitation during the pain. He had no previous history of headache. His medical and family history was otherwise unremarkable. He was not on any medications and used no drugs. His vital signs, a physical examination and a neurological examination were normal. Laboratory testing was normal. His symptoms fulfilled the revised International Classification of Headache Disorders criteria for CH.A diagnosis of CH was made and subcutaneous sumatriptan as well as high-flow oxygen were prescribed. A prophylactic treatment with verapamil was prescribed. He responded to the symptomatic treatment, with relief within 15 minutes. A follow-up was planned. Because the headache attacks continued, the patient was seen again after two weeks. On arrival, a neurological examination was normal. His visual fields were normal. He was taking subcutaneous sumatriptan when required and verapamil 600mg daily. Owing to the relatively late onset of CH, enhanced magnetic resonance imaging (MRI) was ordered to rule out an underlying lesion. It showed a rather large pituitary tumor (20×21×17mm). No clear cavernous sinus invasion could be demonstrated (Figure 1).

The patient had an operation to resect the tumor. Histopathological examination including immunohistochemistry verified the diagnosis of a non-functioning pituitary adenoma (chromophobe adenoma). Hormonal screening showed no abnormalities: pituitary hormones such as prolactin, insulin-like growth factor 1 and growth hormone, thyroid-stimulating hormone and others were within the normal ranges. His headache attacks resolved totally after surgery. He remained headache-free and had not experienced any headache attacks at follow-up after 17 months. The patient is regularly followed up in our endocrine clinic.

## Discussion

This case study involved a patient with CH responding to treatment. An assessment revealed a clinically non-functioning pituitary adenoma. The patient became headache-free after treatment and remained so at follow-up.

Tumors have been documented in patients with CH. The symptoms may be in line with the criteria for CH [3, 4]. Pituitary lesions have been reported within the range of autonomic headache syndromes (trigeminal autonomic cephalalgias). This had led to interest in the link between pituitary tumors and headache [5]. Headache is a common symptom of pituitary disease. Levy *et al*. [5] observed in a study of pituitary tumors and headache that 4% of patients had CH. Functioning adenomas were above all linked to CH. Prolactinomas may be particularly associated with headache. Levy *et al*. [6] described a case of macroprolactinoma associated with CH, which responded to dopamine agonists with complete resolution of the headache. In a reappraisal, Mainardi *et al*. [2] found that pituitary adenomas accounted for 3% of secondary CH cases. The most frequent adenomas were prolactinomas.

The etiology and pathophysiology of CH are unknown. The current hypothesis proposes that primary CH is characterized by hypothalamic activation with secondary activation of the trigeminal-autonomic reflex, probably by a trigeminal-hypothalamic pathway [7]. In this case, the tumor will be non-functioning. The underlying pathophysiology could be due to the structural effects of the tumor itself. Symptomatic CHs due to sellar pathology have also been reported [1, 8]. Olesen *et al*. [7] point to the role of the cavernous sinus and/or hypophyseal region in the pathophysiology of symptomatic CH. The trigeminal nerve may be affected within the cavernous sinus and hypophyseal region by the sympathetic, parasympathetic and sensory fibers of the trigeminal nerve coming together as a plexus in this region [7]. There are patients with pituitary tumor-associated headache who have headache and cranial autonomic symptoms on the same side as the lesion. A local mechanical effect or invasion may be important for the symptoms in those cases [9, 10]. This structural mechanism might have been involved in the pathophysiology of this case.

However, there are studies that contradict this explanation. These studies have failed to show an association between cavernous sinus invasion or tumor volume and headache [9, 11]. Thus, a pituitary tumor-linked headache may not just be a structural problem. It is likely that CH due to sellar pathology has a more multifaceted pathogenesis. The underlying mechanism in these cases remains to be illuminated.

Symptomatic CH should be suspected when the clinical features of the headache are atypical and in patients with pathological findings on neurological examination [7]. Cittadini and Matharu [1] suggest that all patients with CH should be evaluated for pituitary symptoms or disease. They also advocate that enhanced MRI should be carried out in all patients with CH who have an atypical symptomatology, abnormal examination, and a poor response to the appropriate treatments. Many authors propose that neuroimaging should be done in all patients with CH [2, 4, 12]. The motive for this is that symptomatic CH may be indistinguishable from typical episodic CH.

## Conclusions

To the best of the author's knowledge, this is the first report showing an association between CH and a clinically non-functioning pituitary adenoma (chromophobe adenoma). All previous reports have shown an association between CH and prolactinomas or a growth hormone-producing adenoma. CH may be the presenting symptom of a chromophobe adenoma of the pituitary. Symptoms and objective signs of pituitary gland or pituitary region disease are not always present, as in this case. Consequently, the absence of pituitary symptoms or disease does not exclude a symptomatic CH. Tumors have been reported in patients with CH with clinical attacks identical to genuine CH. This case emphasizes the need for imaging procedures in patients with CH. Contrast-enhanced MRI including the sella turcica should always be done in patients with CH.

## Consent

Written informed consent was obtained from the patient for publication of this case report and accompanying images. A copy of the written consent is available for review by the Editor-in-Chief of this journal.

## Abbreviations

CH: cluster headache
MRI: magnetic resonance imaging.
